# A Tribute to Dragan Primorac on the Twentieth Anniversary of The International Society of Applied Biological Sciences and Its Tenth Biennial Conference

**DOI:** 10.3325/cmj.2017.58.201

**Published:** 2017-06

**Authors:** Stanimir Vuk-Pavlović, Moses Schanfield

**Affiliations:** 1 Mayo Clinic College of Medicine and Science, Rochester, Minnesota, USA; vuk@mayo.edu; 2 The George Washington University, Washington, District of Columbia, USA; mschanfield@gmail.com

The Homeland War in Croatia was still far from conclusion when, one day, the phone rang in the office of one of us (SV-P). “I heard you could help me obtain a DNA sequencer,” the voice on the other side said. “We need it to identify the victims from mass graves in Bosnia.” Unfortunately, the help was no more than providing a few connections that later proved useful. At the time, we did not even suspect that twenty-some years later we would write these words.

The voice was of Dragan Primorac, then a postdoctoral fellow at the University of Connecticut. His “I-do-not-take-no-as-the-answer” attitude took him along an unusual and unusually successful trajectory (1). In the intervening years, he became a pediatrician, forensic expert, geneticist, professor, and a hospital founder and director (2). For six years, he had served as the Minister of Science, Education and Sports in Croatian government and was rated as its most successful member. As all highly accomplished people, he sought advice and support from many in the field. His early affiliation with Henry Lee, Michael Baden, one of us (MS), and others introduced him to the most current in forensic genetics. It allowed him to become a pioneer in forensic DNA analysis in Southeastern Europe, with particular success in application of the method to identify bodies from mass graves.

This application has been quite successful as were his other endeavors. He published numerous peer-reviewed and highly cited papers in the most prestigious scientific journals. While teaching at universities of Split and Osijek in Croatia and Pennsylvania State University and the University of New Haven in the United States, Dr Primorac has been the driving force behind the founding, funding, and running The International Society for Applied Biological Sciences (ISABS). Founded initially with the main objective to produce the biennial series of cutting-edge conferences in the areas based on applied genetics, ISABS became a recognized force in the promotion of forensic science and its integration with anthropology and clinical medicine. Among the most recent recognitions of this role, earlier this year the Board of Directors of the American Academy of Forensic Sciences accepted unanimously ISABS as its associate member, the second such organization in its 70-year history (3).

ISABS Conferences are Dr Primorac’s particular achievement. For twenty years, he has bravely pursued his vision of a biennial gathering of scientists of all generations and levels of achievement, from undergraduate students to Nobel Prize winners, to discuss the fields that apply similar or same methods of modern genetic analysis. Starting in 1997 as the European-American Intensive Course in PCR-based Clinical and Forensic Testing, the program nimbly adjusted at each subsequent conference to the developing technology and technological achievements (eg, success of the Human Genome Project). Even more significant has been the introduction into the program of related fields that use the same technology. By virtue of integration of anthropologic genetics/genomics and clinical genetics/genomics, the program became a rather unique cross-section of fields initially related mostly by technology, but today also by the scientific and practical interests. For example, derivation of phenotype from genotype is as much of major interest to forensic science as it is to anthropology and clinical practice.

To maintain the program at the cutting-edge level and fresh, after the Eighth Conference program directors (Dr Primorac and the two of us) passed the baton to the new generation, Manfred Kayser (University of Rotterdam) and Tamás Ördög (Mayo Clinic, Rochester, Minnesota). Affiliation of ISABS with institutions such as Mayo Clinic, American Academy of Forensic Sciences, Pennsylvania State University, and others has never been stronger leading to the ever increasing depth and breadth of the program.

Specific character of the conference series has been recognized by the increasing number of attendees at each subsequent conference (more than 4.000 altogether), presenters (600 in total), and the seventy countries of their origin. Over the years, ISABS has collaborated with the *Croatian Medical Journal* that published special issues containing the total of more than 220 scientific papers presented at conferences (Vol. 42, Nos. 3 and 4, 2001; Vol. 44, Nos. 3 and 4, 2003; Vol. 46, No. 4, 2005; Vol. 48, No. 4, 2007; Vol. 50, No. 3, 2009; Vol. 52, No. 3, 2011; Vol. 54, No. 3, 2013; Vol. 56, No. 3, 2015) (4). Some of these papers have been cited at a respectful rate.

As for the authors of this text, they could not be more grateful to Dragan for having involved them in this project from the very beginning, for sharing credit for its success while carrying all the burden of funding and organizing and, last but not least, for his dedicated friendship.

## References

[1] Dragan Primorac [Internet]. Available from: www.draganprimorac.com. Accessed: June 02, 2017.

[2] St. Catherine Specialty Hospital [Internet]. Available from: www.svkatarina.hr/en. Accessed: June 02, 2017.

[3] International Society for Applied Biological Sciences [Internet]. Available from: www.isabs.hr. Accessed: June 02, 2017.

[4] Croatian Medical Journal. Available from: www.cmj.hr. Accessed: June 02, 2017.

